# Analysis of decisions made in meta-analyses of depression screening and the risk of confirmation bias: A case study

**DOI:** 10.1186/1471-2288-12-76

**Published:** 2012-06-12

**Authors:** Felicity A Goodyear-Smith, Mieke L van Driel, Bruce Arroll, Chris Del Mar

**Affiliations:** 1 Department of General Practice & Primary Health Care, University of Auckland, PB 92019, Auckland 1142, New Zealand; 2 Discipline of General Practice, The University of Queensland, Brisbane, QLD, 4029, Australia; 3 Department of General Practice and Primary Health Care, Ghent University, Ghent, 9000, Belgium; 4 Centre for Research in Evidence-Based Practice, Bond University, Gold Coast, QLD, Australia

**Keywords:** Meta-analysis, Meta-Analysis as Topic, Bias (Epidemiology), Methods, Depression, Mass screening, Social values, Confirmation bias

## Abstract

**Background:**

Depression is common in primary care and clinicians are encouraged to screen their patients. Meta-analyses have evaluated the effectiveness of screening, but two author groups consistently reached completely opposite conclusions.

**Methods:**

We identified five systematic reviews on depression screening conducted between 2001 and 2009, three by Gilbody and colleagues and two by the United States Preventive Task Force. The two author groups consistently reached completely opposite conclusions. We analyzed two contemporaneous systematic reviews, applying a stepwise approach to unravel their methods. Decision points were identified, and discrepancies between systematic reviews authors’ justification of choices made were recorded.

**Results:**

Two systematic reviews each addressing three research questions included 26 randomized controlled trials with different combinations in each review. For the outcome depression screening resulting in treatment, both reviews undertook meta-analyses of imperfectly overlapping studies. Two in particular, pooled each by only one of the reviews, influenced the recommendations in opposite directions. Justification for inclusion or exclusion of studies was obtuse.

**Conclusion:**

Systematic reviews may be less objective than assumed. Based on this analysis of two meta-analyses we hypothesise that strongly held prior beliefs (confirmation bias) may have influenced inclusion and exclusion criteria of studies, and their interpretation. Authors should be required to declare *a priori* any strongly held prior beliefs within their hypotheses, before embarking on systematic reviews.

## Background

Meta-analyses of randomized controlled or N-of-1 trials provides us with the highest level of evidence to inform guidelines and clinical practice. Their validity is therefore important. Over the past 20 years their methodology [1-3] and reporting has been improved. This includes establishing the PRISMA (Preferred Reporting Items for Systematic Reviews and Meta-Analyses) statement (http://www.prisma-statement.org/), which has increased their rigor and transparency. Despite this, however, meta-analyses addressing the same research question arrive at conflicting conclusions or recommendations. The reasons have been explored [4-7]. They include numerous decision points in the review process – such as which study to include or exclude; the risk of bias assessment; and which data to extract. Even within the constraints of a strict protocol, subjective decisions are made.

In this example, we study two different author groups’ meta-analyses of trials investigating the effectiveness of screening for depression, which arrived at opposing recommendations, (one supporting screening and another questioning it) [8-10], in spite of identical research questions.

We wondered about the extent of prior belief in one or other of the possible recommendations, so called ‘confirmation bias’: if an investigator approaches any question with a strong prior belief, their approach to answering that question may be biased [11].

We aim to explore how meta-analyses with the same research question can have opposing recommendations, using a case study approach to examine the decision-points.

## Methods

We chose this example because we were aware of the startling discrepancies in recommendations from different reviews addressing the question. A search was conducted for all systematic reviews and meta-analyses on screening for depression in primary care using the databases MEDLINE, EMBASE,CINAHL, PsycLIT and the Cochrane Database of Systematic Reviews, and hand-searching of the relevant reference lists.

The objectives, findings and conclusions of all accessed reviews were compared (Table1). Two meta-analyses were selected for in depth exploration of the review process. Subsequently, two authors (FGS and MLvD) applied a stepwise approach to unravel the review process followed by the authors of the selected meta-analyses. Each decision moment in the analysis process was recorded alongside an appreciation of the decisions reported by the authors of the selected meta-analyses. Discrepancies between the authors of this study and the justification of choices made were recorded. The two other authors of this paper commented on consistency and transparency of the recorded process and findings. The individual randomized controlled trials (RCTs) included in each review were identified, accessed and examined. A table was constructed recording for each RCT the sample size of the trial, whether or not it favored screening, whether it was included and whether it was pooled in each of the reviews (Table2). The various decisions the authors of the two meta-analyses had made regarding which outcomes to analyze and their data extractions from original studies were explored.

**Table 1:** Comparison of research objectives, findings and conclusions in five reviews

**Reference**	**Objective**	**Findings**	**Conclusion**
Gilbody, 2001 [10]	To examine the effect of routinely administered psychiatric questionnaires on the:	1. Meta-analytic pooling of 4 studies (2457 participants) which measured the effect of feedback on the recognition of depressive disorders found that routine administration and feedback of scores for all patients did not increase the overall rate of recognition of mental disorders such as anxiety and depression.	The routine administration of psychiatric questionnaires with feedback to clinicians does not improve the detection of emotional disorders or patient outcome, although those with high scores may benefit.
	1. recognition,	2. 2 studies showed that routine administration followed by selective feedback for only high scorers increased the rate of recognition of depression.	The widely advocated use of simple questionnaires as outcomes measures in routine practice is not supported.
	2. management, and	3. This increased recognition did not translate into increased rate of intervention.	
	3. outcome of psychiatric disorders in non­psychiatric settings	4. Overall, studies of routine administration of psychiatric measures did not show an effect on patient outcome.	
**Gilbody, 2005**[8]	**To determine the clinical effectiveness of screening and case finding instruments in improving depression:**	**1. According to case note entries of depression, screening/case finding instruments had borderline impact;**	**There is substantial evidence that routinely administered case finding/screening questionnaires for depression have minimal impact on the detection, management or outcome of depression by clinicians.**
	**1. recognition**	**2. Overall trend to showing a borderline higher intervention rate amongst those who received feedback of screening/case finding instruments. This result was dependent upon presence of 1 highly positive study;**	
	**2. management**	**3. 3 out of 4 studies reported no clinical effect at either 6 or 12 months.**	
	**3. outcome.**		
Gilbody, 2008 [9]	To establish the effectiveness of screening in improving the	1. Use of screening or case-finding instruments were associated with a modest increase in the recognition of depression by clinicians	If used alone, case-finding or screening questionnaires for depression appear to have little or no impact on the detection and management of depression by clinicians.
	1. recognition of depression,	2. Questionnaires, when administered to all patients and the results given to clinicians irrespective of baseline score, had no impact on recognition.	Recommendations to adopt screening strategies using standardized questionnaires without organizational enhancements are not justified.
		3. There was no evidence of influence on the prescription of antidepressant medications.	
	2. the management of depression and	4. No evidence of an effect on outcomes of depression was found.	
	3. the outcomes of patients with depression.		
**USPTF, 2002**[12,13]	**1. What is the accuracy of case-finding instruments for depression in primary care populations?**	**1. Compared with usual care, feedback of depression screening results to providers generally increased recognition of depressive illness in adults.**	**Compared with usual care, screening for depression can improve outcomes, particularly when screening is coupled with system changes that help ensure adequate treatment and follow-up.**
	**2. Is treatment of depression in primary care patients effective in improving outcomes?**	**2. Studies examining the effect of screening and feedback on treatment rates and clinical outcomes had mixed results. Many trials lacked power to detect clinically important differences in outcomes.**	
	**3. Is routine systematic identification with case-finding questions (screening), with or without integrated management and follow-up systems, more effective than usual care in identifying patients with depression, facilitating treatment of patients with depression, and improving clinical outcomes?**	**3. Meta-analysis suggests that overall, screening and feedback reduced the risk for persistent depression.**	
		**4. Programs that integrated interventions aimed at improving recognition and treatment of patients with depression and that incorporated quality improvements in clinic systems had stronger effects than programs of feedback alone.**	
USPTF, 2009[14,15]	To review the benefits and harms of screening adult patients for depression in a primary care setting	1. Primary care depression screening and care management programs with staff assistance, such as case management or mental health specialist involvement, can increase depression response and remission.	1. The USPSTF recommends screening adults for depression when staff-assisted depression care supports are in place to assure accurate diagnosis, effective treatment, and follow-up. (Grade B recommendation)
		2. Benefit was not evident in screening programs without staff assistance in depression care.	2. The USPSTF recommends against routinely screening adults for depression when staff-assisted depression care supports are not in place. There may be considerations that support screening for depression in an individual patient. (Grade C recommendation)

**Table 2:** Comparison of trials included and pooled in 5 systematic reviews of depression screening

**Reference**	**N**	**Favors screening**	**Gilbody, 2001**[10]		**Gilbody, 2005**[8]		**Gilbody, 2008**[9]		**USPTF, 2002**[12,13]		**USPTF, 2009**[14,15]	
**Search date**			**2000**		**2004**		**2007**		**2001**		**2007**	
			**Incl**	**Pool**	**Incl**	**Pool**	**Incl**	**Pool**	**Incl**	**Pool**	**Incl**	**Pool**
Johnstone, 1976 [16]	1093	++	yes	no	no	no	yes	no	yes	no	yes	no
Moore, 1978 [17]	212	+	yes	yes	yes	yes	yes	yes	yes	no	yes	no
Linn, 1980 [18]	150	+	yes	no	yes	yes	yes	yes	yes	no	yes	no
Zung & Magill, 1983 [19]	143	++	no	no	yes	no	yes	no	no	no	yes	no
Zung & King, 1983 [20]	49	++	no	no	no	no	no	no	yes	yes	no	no
Hoeper, 1984 [21]	1452	-	yes	yes	yes	yes	yes	yes	no	no	no	no
German, 1987 [22]	488	+	yes	yes	yes	yes	yes	yes	no	no	no	no
Magruder-Habib, 1990 [23]	100	++	yes	yes	yes	yes	yes	yes	yes	no	yes	no
Callahan, 1994 [24]	175		no	no	yes	yes	yes	no	yes	yes	yes	no
Dowrick, 1995 [25]	179	-	yes	no	yes	yes	yes	yes	yes	no	yes	no
Callahan, 1996 [26]	222	-	no	no	no	no	yes	yes	yes	yes	yes	no
Lewis, 1996 [27]	681	+	yes	no	yes	yes	yes	yes	yes	yes	yes	no
Mazonson, 1996 [28]	573	++	yes	no	no	no	no	no	no	no	no	no
Reilfer, 1996 [29]	358	+	no	No	no	no	no	no	yes	no	no	no
Williams, 1999 [30]	969	+	no	no	yes	yes	yes	yes	yes	yes	yes	no
Katzelnick, 2000 [31]	407	++	**no**	**no**	no	no	no	no	yes	yes	yes	no
Weatherall, 2000 [32]	100	-	**no**	**no**	yes	yes	yes	yes	no	no	no	no
Wells, 2000 [33]	1356	++	**no**	**no**	no	no	no	no	yes	yes	yes	no
Whooley, 2000 [34]	331	-	**no**	**no**	yes	yes	yes	yes	yes	yes	yes	no
Rost, 2001 [35]	479	++	**no**	**no**	no	no	no	no	yes	no	yes	no
Schriger, 2001 [36]	218	-	**no**	**no**	no	no	yes	yes	**no**	**no**	no	no
Christensen, 2003 [37]	1785	+	**no**	**no**	no	no	yes	yes	**no**	**no**	no	no
Jarjoura, 2004 [38]	61	++	**no**	**no**	no	no	no	no	**no**	**no**	yes	no
Bergus, 2005 [39]	51	-	**no**	**no**	**no**	**no**	yes	yes	**no**	**no**	no	no
Bosmans, 2006 [40]	145	-	**no**	**no**	**no**	**no**	no	no	**no**	**no**	yes	no
Rubenstein, 2007 [41]	792	+	**no**	**no**	**no**	**no**	**no**	**no**	**no**	**no**	yes	no

N = Total number in the trial at baseline (control and intervention arms).

Incl = Included for any or all of three outcomes (recognition, management, outcome of depression).

Pool = Pooled for any or all of three outcomes (recognition, management, outcome of depression).

Cells shaded where study was not available for review with that search date.

USPTF = US Preventive Task Force.

## Results

**Figure 1:**
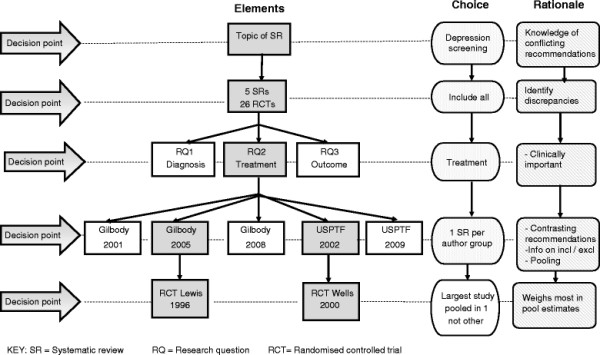
Flowchart of decision points and rationale for choices when comparing contrasting systematic reviews.

The results of our explorative analysis are presented in the flowchart (Figure1). Five systematic reviews (four with pooled data) were identified. Three meta-analyses were conducted by Gilbody and colleagues between 2001 and 2008, including one Cochrane review [8-10]. None of these favoured screening. Two reviews (one meta-analysis) from another author group, the US Preventative Task Force (USPTF), in 2002 [12,13] and 2009 [14,15] favoured screening (Table1).

The five reviews included a total of 26 RCTs [16-41] and not one of these was included in all reviews (Table2). For example, for the outcome of providing practitioners with feedback on screening (detection of possible depression) prior to initiation of treatment, Gilbody 2001 [10] pooled four RCTs [17,21-23] whereas for the same outcome the USPTF pooled a completely different set of seven RCTs.[20,26,27,30,31,33,34] All of these studies would have been available to both author groups with the exception of the study by Wells [33], which might not have been published when Gilbody et al. conducted their search.

Each of the five reviews considered three different research questions (effectiveness on detection, treatment and patient outcomes) with different combinations of RCTs included for each. Again, none of these were common between reviews. This meant that there were 15 different combinations of RCTs for the five reviews considering the three research questions. For pragmatic reasons we decided to select two reviews with opposing recommendations which addressed the same research question to determine factors leading to discrepant findings.

The two meta-analyses we selected for comparison, one favouring and the other not favouring screening were the Cochrane review by Gilbody of 2005 [8] and the USPTF 2002 meta-analysis [13]. These two meta-analyses contained the most information on both included and excluded trials, had the most overlapping studies and both included pooled data. We decided to focus on only one of the three research questions addressed in the meta-analyses. The outcome of the effect of depression screening on treatment (i.e. if the patient received treatment for depression) was selected because this is of clinical importance and also included the largest number of studies used in the reviews. We identified RCTs included and pooled in either review and then examined these to determine which most influenced the results favouring screening or not screening.

We found that the opposing recommendations of the two reviews were largely determined by the Lewis study [27] pooled in the Cochrane but not the USPTF review, and the Wells trial [33] pooled in the USPTF but excluded from the Cochrane review.

On inspection of the forest plot in the Cochrane review for the outcome of management of depression following feedback (prescription of anti-depressants) [8] (their Analysis 2.2, p 28), the Lewis study [27] has the greatest weighting (37.5%). It can be seen clearly that this study shifts the plot from favoring screening to favoring not screening. The USPTF included this study in their review but did not pool it for this outcome because they report that the figures *“cannot be calculated from available data”*. There were 227 patients in each of the control and screened arms. The Cochrane review has entered the Lewis study in their forest plot as 100/227 for control and 125/227 for screening. It is unclear how they have derived these numbers. The Cochrane review states that for the Lewis study they used published data only [8]. The Lewis study reports that the mean number of psychotropic drug prescriptions for the control arm was 0.44 (SD 1.58) and for the screened arm was 0.55 (SD 1.43) with a *p* value of 0.6 (their Table 5) [27]. However the mean number of drugs prescribed does not necessarily equate to the proportion of patients taking psychotropic drugs. Our own attempts to contact the authors of the Lewis paper to obtain their data have been unsuccessful to date.

The RCT in the USPTF review [13] which has the greatest weighting and clearly influences the finding favouring towards screening is the Wells study.[33] This study enrolled 1356 patients who were screened as depressed using the “stem” items for major depressive and dysthymic disorders from the Composite International Diagnostic Interview (CIDI) [33]. Randomization was by clinic which either provided usual care (provider not informed that their patients were in the trial) or provided a quality improvement program with either psychotropic medication or psychological intervention (providers notified that their patients had screened positive for depression). The quality of care, mental health outcomes and retention of employment of depressed patients improved in the intervention group. The Wells study is excluded from the Cochrane review because it is a *“Complex quality improvement programme”* (Characteristics of excluded studies, p22) [8].

## Discussion

What initially presented as a straightforward task revealed itself to be increasingly complex when we discovered that in the five reviews each considering three outcomes, there were 15 different combinations of RCTs. Our analysis of the process of two meta-analyses that address the same research question but reach contradictory conclusions demonstrates how decisions in the meta-analysis process can shape the conclusion. This is an important finding as evidence-based clinical guidelines and practice recommendations rely on evidence from systematic reviews and meta-analyses.

Two questions come to mind; “Who is right?” and, “What drove the decisions?" The second question is the most essential one that requires full attention from meta-analysts. Addressing the fundamental issue of human choices in a methodologically rigorous process might even make an answer to the first and most intuitive question superfluous.

There is ample literature on the impact of publication bias, referring to an overrepresentation of trials with a ‘positive’ outcome in searches, on the conclusions of meta-analyses [4,42]. This type of bias can be addressed by searching for unpublished data or extending the search to languages other than English [2], although it is not clear if this is worth the effort [43].

Discrepancies in outcomes of meta-analyses have been documented and are often attributed to selective inclusion of studies [5,44,45]. Felson describes a model for bias in meta-analytic research identifying three stages at which bias can be introduced: finding studies, selection of studies to include and extraction of data.[46]. He argues that *“selection bias of studies* [as opposed to selection bias of individuals within studies] *is probably the central reason for discrepant results in meta-analyses.*” Cook et al. determined that discordant meta-analyses could be attributed to *“incomplete identification of relevant studies, differential inclusion of non-English language and nonrandomized trials, different definitions .., provision of additional information through direct correspondence with authors, and different statistical methods”*[47]. Another study of eight meta-analyses found *“many errors in both application of eligibility criteria and dichotomous data extraction*” [48].

While selection bias and differing data extraction may contribute to discrepancy, our study suggests that the bias begins before these steps. Over three research questions in five different reviews, we found 15 different sets of RCTs were included, yet one author group consistently found against while the other found for screening. Even though the two systematic reviews have cited each other’s earlier publication this does not appear to have prevented the discrepancies. Which studies are included and which data from these studies are used involves numerous decisions. To our knowledge, the issue of choices and decision making in the process of meta-analysis has not been studied empirically before.

The methodology of meta-analysis is well developed and is continuously being refined to address identified threats of bias. The process is well documented in numerous text books, of which the Cochrane Collaboration Reviewers’ Handbook [2] may be the most widely used. The Cochrane Collaboration, the largest database of systematic reviews and meta-analyses of clinical trials in medicine, requires its authors to produce a protocol describing the intended process of the review before embarking on the review. Each step is peer reviewed and monitored by editorial groups, ensuring methodological rigor. But no matter how rigorously we describe each step in the process, human decisions are being made all the time. When documenting each decision we made in our exploration, we ourselves, although experienced reviewers, were astonished by the number of decision moments that occurred. Moreover, some of these decisions could be traced to ‘subjective’ inclinations. For example, our choice to explore the question related to effect of screening on number of patients on treatment, was based on a compromise of the desire to study a clinically relevant question and at the same time have enough material for further study. Documenting each of these decisions and the rationale for the choices could add transparency to the process.

However, there might be an even more fundamental implicit source of “bias” embedded in the review process. The consistent findings of the two author groups suggests this. We hypothesise that authors may have a belief of what the outcome of their meta-analysis will be before they start, and that this belief may guide choices that are made on the way which may impact the review's results. This is a form of confirmation bias [49,50].

This could be an important first form of bias in the complex decision process of a meta-analysis. It refers to the fact that authors or researchers seek or interpret evidence in ways that fit with (their own) existing beliefs, expectations, or hypothesis [49]. Confirmation bias has many different aspects according to the context in which it is analysed and been shown to play a role in clinical decision making [50], but to our knowledge it has not been applied to risk of bias assessment of meta-analyses. Unravelling this concept and making its impact explicit in the meta-analysis process could contribute to a better understanding of (often implicit) forms of bias that guide the reviewers’ choices along the way.

Meta-analyses with different conclusions may result in opposing recommendations with important consequences which might be reflected in clinical guidelines, as is the situation in our case, where the US guidelines recommended screening but the UK ones recommended not screening. We recommend that guideline writers and health policy makers should check all available systematic reviews to ensure such discrepancies do not exist. Where contradicting reviews are found guideline writers should address these discrepancies and justify any stand they take, not make a subjective decision to suit their own pre-conceived beliefs. This is where prior disclosure of belief of what the outcome will be would be of assistance.

The main limitation of our study is that we chose to compare only two meta-analyses from the many options available and we have introduced subjectivity by the choices we made. However, making these choices and their potential subjectivity explicit is the main strength of the study. Our proposal of confirmation bias to explain the dissonance can only be a hypothesis. It requires further study, comparing and unravelling decision points in other meta-analyses .

## Conclusion

No meta-analysis is value-free. PRISMA involves a 27-item check list (http://www.prisma-statement.org/), and expanding this would not solve the problem of confirmation bias. Nevertheless, we were surprised at the number of decision points in a meta–analysis, and propose an additional step of recognising each decision point and being explicit about these choices and their rationale would greatly increase the transparency of the meta-analysis process. But a better improvement in transparency of meta-analysis could perhaps be achieved by asking authors to declare their belief of the outcome before they embark on the review process. This step can easily be built into the review process of the Cochrane Collaboration, where the review protocol precedes publication of the full review. The implicit “subjectivity” of the seemingly “objective” meta-analysis process deserves attention in all published reviews and is an important part of well-informed evidence-based practice.

## Competing interests

The authors declare that they have no competing interest.

## Authors’ contributions

FG-S conceived of the study, participated in its design and coordination, analysis and helped draft the paper. FG-S had full access to all the data in the study and takes responsibility for the integrity of the data and the accuracy of the data analysis. MLvD assisted in the study design, analysis and writing of the paper. BA was involved in initial conception of the study and contributed to the analysis and writing of the paper. CDM suggested subsequent study analyses and made critical additions to the manuscript. All authors read and approved the final manuscript.

## Funding

Nil
